# Preparation of β-Cyclodextrin(CD)/Flavour CD Powder and Its Application on Flavour Improvement of Regular Coffee

**DOI:** 10.3390/foods13152359

**Published:** 2024-07-26

**Authors:** Zhiheng Zhang, Haicheng Liang, Zichun Chai, Ting Wang

**Affiliations:** 1 Aulin College, Northeast Forestry University, Harbin 150040, China; zhang040503@nefu.edu.cn (Z.Z.); hlia974@aucklanduni.ac.nz (H.L.); chai020304@nefu.edu.cn (Z.C.); 2 Food Science Programme, School of Chemical Sciences, University of Auckland, Auckland 1010, New Zealand; 3 Feixiang Technology Service (Harbin) Co., Ltd., Harbin 150040, China; 4 College of Chemistry, Chemical Engineering and Resource Utilization, Northeast Forestry University, 26 Hexing Road, Harbin 150040, China

**Keywords:** β-CD/flavour CD powder, volatile compound, coffee aroma analysis, sensory evaluation, aroma and flavour

## Abstract

To improve the overall sensory evaluation of regular coffee, a mixture of β-CD/flavour CD powder was prepared by a freeze-drying method. Cyclodextrin inclusion complexes consist of eight compounds that are naturally present in coffee, specifically: 2,5-dimethylpyrazine, benzaldehyde, citral, linalool, limonene, phenethyl acetate, furfural, and ethyl acetate. These eight compounds naturally occur in coffee, making them safer than using other compounds. Moreover, these eight compounds are the primary active ingredients in coffee, significantly influencing its flavour profile. Therefore, choosing to complex these eight compounds with cyclodextrins can effectively enhance the taste of the coffee. XRD, FT-IR, and SDE-GC-FID were presented to study the formation of inclusion CD powder, the storage stability, chemical composition changes, and safety. Results show that by the cyclodextrin method of freeze-drying, the CD powder showed a stable encapsulated structure and increased stability of flavour compounds. Based on the coffee aroma analysis results, prepared CD powder can enhance the coffee’s aroma score by 3.0–4.0 points and increase the flavour score by 2.1–3.5 points, and it can achieve preservation for a minimum of 181 days at 25 °C. Furthermore, under the requirements of the China national standard for additives, the mixture of β-CD/flavour CD powder was used for the cup testing with four regular coffees to obtain improved coffees. With the full score is 10, improved coffees could score extra 3.0–4.0 points on aroma and 2.1–3.5 on flavour compared to regular coffee. In addition, the CD powder also improves the quality of the coffee in terms of aftertaste, body, and sweetness. Overall, β-CD/flavour CD powders provide several advantages over the currently popular coffee bean processing methods, including improved reproducibility, enhanced controllability, and increased flexibility, while prioritizing safety. And it should be explored further with appropriate compounds given its potential for coffee aroma modulation.

## 1. Introduction

The scale of coffee consumption in China is expanding, with a year-on-year growth rate of around 30%, and specialty coffee is favoured by more and more consumers for its unique aroma and flavour [1,2]. Aroma components are especially vital in coffee because they are the main components of the sensory experience of coffee [3,4]. Those aroma components are the product of Maillard and the other reactions [5,6]. Even though the conditions for the Maillard reaction are simple, the extent of the reaction is hard to control [7]. Therefore, preserving the compounds responsible for coffee’s distinct flavour and aroma during reactions is challenging [8]. In consequence, finding a convenient and effective way to improve coffee flavour and aroma is of great significance to enhance the commercial value of coffee [9,10,11].

Traditionally, based on the purpose of improving cupping quality, the research on chemical composition in coffee has become the focus of extensive attention [12]. There are two traditional ways to treat green coffee beans: the natural process and the washed process [13]. In the natural process, green coffee beans are naturally dried in their flesh, and they are allowed to mature naturally inside the fruit, so naturally processed coffee beans are fruity, sweet, and distinctly mellow [14]. The washed process was invented due to the excessive disadvantages of the natural process [15]. The coffee beans treated by the washed process have a clean flavour, bright acidity, fresh taste, and a sense of fermentation [13]. However, it is impossible to achieve optimal flavour for all green beans only using these two traditional methods. 

Recently, research has been conducted on fermentation technology, and the application of fermentation technology to process green coffee beans has become a hot topic [16]. Since the middle of the 1900s, a mass of species of microorganisms have been isolated from the fermentation phase of wet processing [17]. Benefax, Pectozyme, Cofepec, and Ultrazym are commercially available enzymes designed for coffee fermentation [18]. Evangelista utilized yeast strains *C. parapsilosis* UFLA YCN448 and *S. cerevisiae* UFLA YCN727, producing coffee with special aroma of caramel, herbs, and fruits [19]. Because the current edible enzyme production enterprises are still mainly using outdated equipment, it is difficult to perform large-scale production [20]. Furthermore, in the processing by enzymes, impurities can easily be introduced, and the quality of the coffee can be seriously affected [21]. Ribeiro [19] selected types of mesophilic and lactic acid bacteria strains for compound fermentation. However, in the case of multi-bacteria co-fermentation, the indicators of fermentation are not fixed, and it is difficult to control; in addition, the fermentation time is usually very long [20]. Moreover, unpleasant taste and toxic miscellaneous bacteria could be introduced into the green coffee beans by over-fermentation and unqualified sterilization. Therefore, based on the fermentation technology, better coffee flavour and aroma improvement techniques still need to be studied.

Cyclodextrins (CDs) are a group of biocompatible and non-toxic α-(1,4)-oligosaccharides derived from starch. They consist of six (α), seven (β), or eight (γ) glucose units and are widely recognized for their role as encapsulating agents [22]. Due to their cage-like supramolecular structure, they are also used as a nanocarrier [23]. Among them, β-Cyclodextrin (β-CD) is a cyclic oligosaccharide made up of seven β-pyranose units. It is a small molecule derived from natural starch, produced by the enzymatic action of cyclodextrin glucosyltransferase on starch [24]. It is formed through α-1,4-glycosidic bonds that link glucose units together, resulting in a rigid conical cavity structure with a hydrophobic interior and a hydrophilic exterior [25]. Its energy is enough to match a few guest molecules of polarity, size, shape, and nature or the hydrophobic groups of some guest molecules [26]. β-CD is widely used in food and pharmaceutical fields due to its availability [27], moderate cavity size, and low cost, which can encapsulate substances with low and high molecular weights [28]. The inclusion by β-CD can also improve the stability, water solubility, and bio-availability of the guest compounds [29] and ensure sustained release of guest compounds from the inclusion compound [30,31]. There are mainly the following aspects for application of β-CD in coffee: (1) Protecting sensitive substances and volatile components in raw materials. (2) Producing high aroma and quality in instant drinks [32]. (3) Eliminating the bitter taste in coffee through the use of cyclodextrins [33,34]. Therefore, by utilizing cyclodextrins to complex with other compounds to create coffee flavour enhancers, we can not only mitigate the bitterness in coffee but also enhance its aroma on this basis, thereby achieving the goal of refining the coffee’s taste profile. It is noteworthy that β-CD not only exhibits a beneficial protective effect on the volatile compounds that contribute to coffee flavour but also has the ability to form complexes with migratory bitter compounds such as monocaffeoyl quinic acids, dicaffeoyl quinides, and 4-vinylcatechol in dry powder mixtures, utilizing the water of crystallization present in cyclodextrin oligomers [35,36,37,38].

This study is based on the coffee aroma analysis experiment [16,39,40,41]. To improve the aroma and flavour of coffee, linalool, D-limonene, 2,5-dimethylpyrazine, ethyl acetate, phenethyl acetate, citral, furfural, and benzaldehyde are chosen [42,43] to prepare an inclusion compound with β-CD, and a mixture of β-CD/flavour was prepared and characterized [34]. Then, this flavour powder complex was added to regular coffee. Sensory evaluation experiments were performed by several SCA baristas and well-trained laboratory personnel according to SCA standards.

## 2. Materials and Methods

### 2.1. Materials

Four types of roasted coffee beans (sinloy yixia#1, sinloy landong#2, sinloy yishijiaotang#3, and sinloy mandheling#4) were purchased from Baoshan Zhongka Food Co., Ltd. (Baoshan, Yunnan, China), and all four varieties of coffee beans are Arabica coffee beans. Ethyl acetate (FG), citral (FG), phenethyl acetate (FG), furfural (FG), linalool (FG), limonene (FG), benzaldehyde (FG), 2,5-dimethylpyrazine (FG), and isoamyl acetate (AR) were all purchase from Sigma-Aldrich (Shanghai) Ltd. (China). β-CD (FG), dichloromethane (AR), and absolute ethanol (AR) were all purchased from Aladdin Co., Ltd. (Shanghai, China).

### 2.2. Preparation of β-CD/Flavour CD Powder

**Figure 1:**
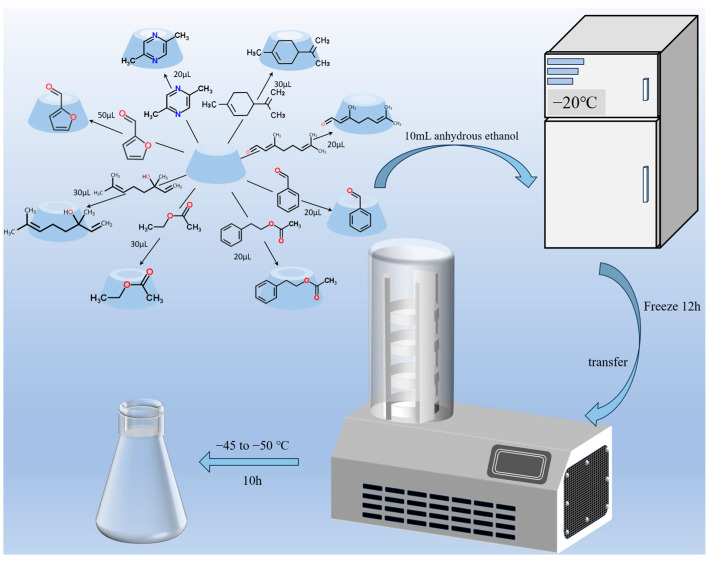
The structural relationship of cyclodextrin with the eight compounds.

Previous studies were adapted to prepare β-CD/flavour CD powder. The optimal experimental material ratios, reaction temperatures, and reaction time were obtained by conducting comparative experiments. Firstly, 1.85 g of β-CD was accurately weighed, dissolved in distilled water, and heated and stirred in a water bath at 60 °C until completely dissolved. The coffee flavour substances include 20 μL of 2,5-dimethylpyrazine, 20 μL of benzaldehyde, 20 μL of citral, 30 μL of linalool, 30 μL of limonene, 20 μL of phenethyl acetate, 50 μL of furfural, and 30 μL of ethyl acetate, and they were added into 10 mL of anhydrous ethanol, respectively, and then mixed together. Following that, the mixture was introduced into the previously mentioned solution and stirred for 2 h at 20–25 °C. Subsequently, the mixture was allowed to stand in a refrigerator at −20 °C for 12 h before being transferred to a vacuum freeze-dryer equipped with a cold trap set at −45 to −50 °C for a freeze-drying period of 10 h. The resulting dried powder represents the desired final product [44,45]. The structural relationship of cyclodextrin with the eight compounds is shown schematically in Figure 1.

### 2.3. Characterization

#### 2.3.1. Determination by FT-IR

A Nicolet IS 10 (Thermo Fisher Scientific Co., Ltd., Shanghai, China) infrared spectrometer was used for the experiment. Analysis of eight kinds of coffee flavour substances, β-CD, and β-CD/flavour CD powder was performed using an FT-IR spectrometer in the mid-infrared region in the wave number range of 4000 to 400 cm^−1^ with a spectral resolution of 4 cm^−1^.

Prior to measurement, the solid samples of β-CD and β-CD/flavour CD powder were ground and mixed thoroughly with KBr. Then, the mixed samples were compressed in a tablet press to make KBr tablets, which were then placed in the sample slot of the FT-IR spectrometer for scanning and analysis.

#### 2.3.2. Determination by XRD

The sample of β-CD, physical mixture of β-CD, and β-CD/flavour CD powder were weighed and ground appropriately. And they were tested using a PANalyticalX’Pert Powder diffractometer. The scattering slit was set to 1/4°, and the evanescent slit was set to 1/8°. The tube voltage and current were, respectively, set to 40 kV and 40 mA, and the test was performed in a 2θ angle range between 5° and 30°. The scan rate was set to 1.5° min^−1^, patterns for the samples were collected by using Cu Kα radiation, and the data were saved for subsequent analysis.

### 2.4. Flavour Determination by SDE-GC-FID

Chromatographic conditions: the inlet sample temperature was 250 °C, and column ramp-up progress was as in Table 1. The flow rate of N_2_ carrier gas was controlled at 1 mL min^−1^, the split ratio was 80:1, and the injection volume was 0.4 μL.

Four experimental coffee beans were precisely weighed, with each sample weighing 20.0 g, and the coffee beans are roasted. These beans were placed in a round-bottom flask along with 348 mL of distilled water. One end of the simultaneous distillation extraction device was connected to the flask, while 30 mL of dichloromethane was added to the round-bottom flask at the other end of the extraction device. The mixture was heated using an electric furnace for a duration of 3 h. Subsequently, the extracts were dried overnight using anhydrous sodium sulphate and concentrated to 5 mL using a rotary evaporator, ready for GC-FID analysis.

GC-FID analysis was conducted using an Agilent 7890A gas chromatograph (Agilent Technologies Inc Co., Ltd., Beijing, China). A total of 1.85 g of the β-CD/flavour CD powder inclusion compound was weighed and combined with 10 mL of methanol. The mixture underwent ultrasonic extraction for 20 min, followed by centrifugation at 5000 rpm for 10 min. The resulting supernatant was collected for GC analysis.

### 2.5. Storage Performance Test of the Inclusion Compound

#### 2.5.1. Storage Stability

To evaluate the release of coffee aroma substances encapsulated in the inclusion complex under normal storage conditions, a certain amount of 1.85 g β-CD/flavour CD powder (encapsulated with an equal amount of coffee aroma substance) was placed in a Petri dish and then stored in a vacuum desiccator at 20–25 °C (ambient temperature). The mass loss of the coffee aroma substance was recorded for 30 consecutive days. The content of coffee aroma substances retained in the β-CD/flavour CD powder was further determined by GC-FID under the conditions described above. 

#### 2.5.2. Accelerated Destructive Test

An accelerated destruction experiment (ASLT) model was used to determine the retention time of 1.85 g of β-CD/flavour CD powder cyclodextrin inclusion complex. The shelf life of the inclusion compound at a certain storage temperature can be predicted by increasing the storage temperature to accelerate the deterioration [46]. The shelf life is recorded at two temperatures of 37 °C and 47 °C, respectively. The ratio of the two shelf lives of the two temperatures is calculated. Then, the required β-CD/flavour CD powder coffee flavour inclusion compound shelf freshness is predicted according to Equation (1).

(1) $$\frac{Q_{10}(T_{0}-T)}{10}=\frac{Q_{S}(T)}{Q_{S}(T_{0})}$$

where *Qs* is the shelf life (days), $Q_{S}(T)$ is the shelf life measured in the higher-temperature (T) experiment, $Q_{S}(T_{0})$ is the shelf life of the lower-temperature ($T_{0}$) experiment, and *Q*_10_ is the ratio of shelf life with a temperature difference of 10 °C.

Sensory assessments were also conducted on coffee sample #1 every 5 days by three Specialty Coffee Association-certified intermediate baristas. The accelerated destructive test may be terminated if there have been significant and unacceptable changes in the senses or indicators.

### 2.6. Sensory Test

The sensory test followed the standard of the Specialty Coffee Association [47]. Three SCA-certified intermediate baristas and some well-trained coffee tasters are organized for this study. Our sensory test participants underwent a comprehensive training programme led by SCA-certified intermediate baristas. The training included theoretical knowledge on coffee types, origins, treatment methods, and SCA’s ten dimensions of sensory analysis. Participants learned sensory vocabulary and cup testing methods. Sensory calibration involved using a 36-flavour smelling kit and aqueous solutions to calibrate aroma, flavour, acidity, and sweetness, followed by tasting various coffees to calibrate attributes like aftertaste and body. Finally, a discussion session allowed participants to share experiences, compare sensory descriptions, and address common issues under the guidance of the trainer. They all underwent sensory calibration before the test. Four types of regular coffees and improved coffees are prepared for the test. And two methodologies were applied. Before the commencement of the experiments, to ensure consistency between the two sets of coffee used, all varieties underwent a duplicate uniformity test. Prior to the start of the experiment, the homogenized coffee was divided into two portions, each sample of which was cupped by three SCA-certified intermediate baristas who determined if the two samples were identical in taste and flavour. Through our testing process, we ascertained that the content of effective flavour components in both coffee samples was consistent. Firstly, participants began the evaluations when the time of tasting was of 4 min after mixing; this is to ensure that the coffee fully mixes with the CD powder during this time, to guarantee homogenization of the mixture. At a ratio of 1.63 g of coffee per 1 fluid ounce of water, 14 g of coffee beans and 255 mL of water were prepared. Then, 0.3 g of β-CD/flavour CD powder was added to obtain improved coffee. The infusion point of water was performed after the water reached 92.2–94.4 °C. It is necessary to prepare specified spoons for cupping which shall hold 0.135–0.169 fluid ounces (4–5 mL) of coffee sample and should be of non-reactive metal. Fragrance/aroma, flavour, aftertaste, acidity, body, balance, sweetness, clean cup, uniformity, and overall are the variables used in the descriptive sensory analysis by SCA protocol. After cupping 8 cups of coffee, SCA Arabica Cupping Forms and statistic data were collected. 

For the other testing, a double-blind experiment was used. The experiment was divided into eight 10 min sessions. Four types of coffee beans were used to brew eight cups of coffee, four of which were regular coffee and four of which were our improved coffees. During the test, the participants did not know the variety of coffee, did not know if the coffee was improved, were not able to communicate with other tasters, and tasted the coffee within the limited time and filled out the SCA Arabica Cupping Form. During the whole process, coffee makers, delivery personnel, and participants were all independent units. All participants had to give a mark on each of the eight mentioned aspects. The scores of those 8 monitoring points range from 1 to 10, respectively. And in these two experiments, the amounts of ground coffee, complex CD, and water used in the second sensory test were consistent with those in the previous sensory test, and they yielded similar results.

## 3. Results and Discussion

### 3.1. Characterization of β-CD/Flavour CD Powder

**Figure 2:**
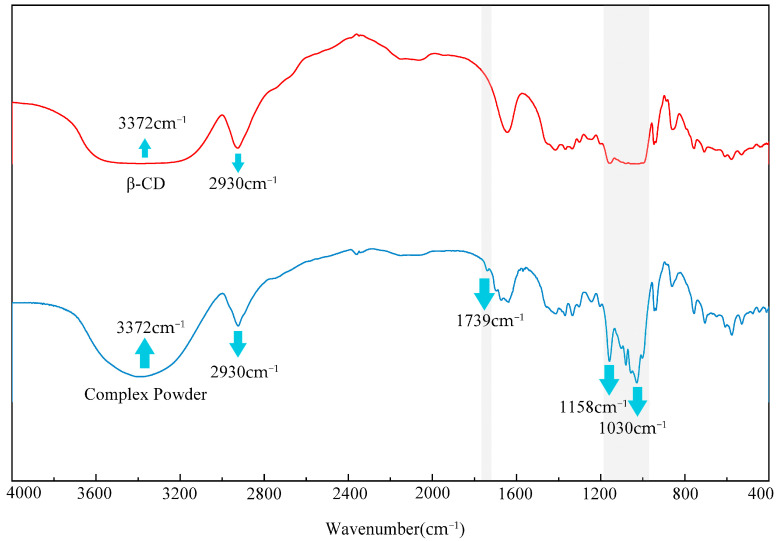
The characteristic peak of FT-IR spectra of β-CD and CD powder.

The inclusion interaction between the compounds and β-CD was further investigated using FT-IR analysis. The results are shown in Figure 2. In this study, we determined the FT-IR spectra of β-CD and the CD powder from 4000 to 400 cm^−1^ [48], and the formation of the inclusion complex was verified by the difference in peak positions of the spectra [49]. As shown in Figure 2, as well as the 3372 cm^−1^ and 2930 cm^−1^ typical peaks of β-CD, the CD powder has obvious additional peaks at 2360 cm^−1^, 1739 cm^−1^, 1158 cm^−1^, and 1030 cm^−1^ ascribed to encapsulated flavour substances. 

**Figure 3:**
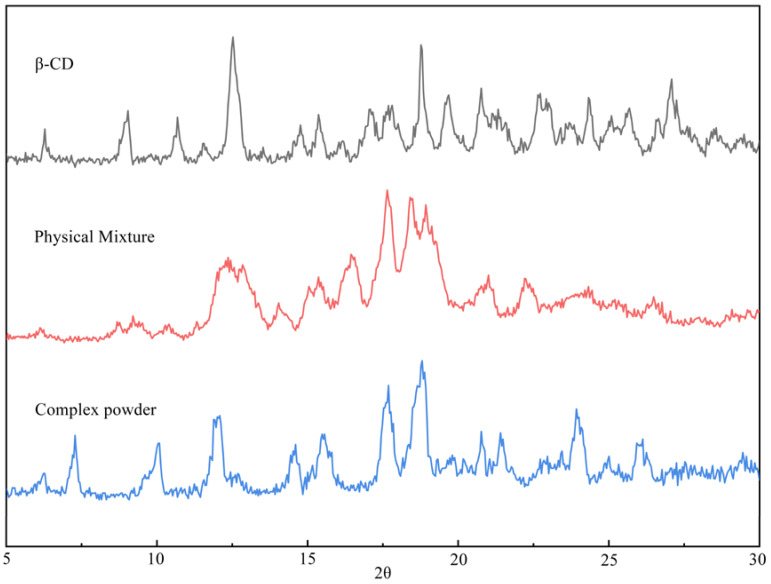
XRD spectra of β-CD, physical mixtures, and CD powder.

The inclusion complex formation was also verified by XRD, which has been proven to be an effective method for the analysis of inclusion complexes [50,51]. Through the results of our experiments in Figure 3, it can be seen that the XRD diffraction patterns of the β-CD inclusion complex are significantly different from those of pure β-CD and physical mixtures; the appearance of the new peaks is a result that suggests the formation of coffee aroma substance/β-CD inclusion [52,53,54]. The plots of β-CD powders have some intense and sharp peaks at diffraction angles of 2θ of 9.04°, 10.68°, 14.76°, 15.40°, 17.08°, 18.76°, 20.75°, 25.64°, and 27.08°. This suggests that the powder is present as a crystalline material [55,56]. The XRD spectra of the physical mixture of flavour components and β-CD show some typical peaks of β-CD that may be attributed to the crystalline structure, but the intensity of the peaks changes considerably, which indicates that the crystallinity of β-CD has undergone changes due to the influence of the compound. In contrast, the diffraction pattern of the inclusion complex is different from the superimposed pattern of the main and guest components. The XRD pattern of the β-CD/flavour CD powder shows the disappearance of some of the original crystalline peaks, and some new sharp peaks are observed at 2θ of 7.24°, 10.12°, 14.44°, 15.56°, 17.68°, and 4.16°, respectively [57].

### 3.2. Analysis of β-CD/Flavour CD Powder

The type and content of each compound were detected by GC. The quantitative analysis of the components was carried out by GC-FID using the internal standard method, and the results are shown in Table 2. A total of eight compounds were detected: benzaldehyde, citral, furfural, ethyl acetate, phenylethyl acetate, limonene, linalool, and 2,5-dimethylpyrazine. By calculating the content, the aldehydes in the powder release of the inclusion compound accounted for 2.84%, of which benzaldehyde made up 0.55%, citral 0.71%, and furfural 1.58%. Esters accounted for 1.55%, of which ethyl acetate made up 0.98% and phenethyl acetate 0.57%. Limonene accounted for 0.64%, linalool accounted for 0.62%, and 2,5-dimethylpyrazine accounted for 0.87%. In the CD powder, β-CD accounts for the highest proportion at 93.48%.

All usage of the eight compounds is permitted by the standard for the usage of food flavour varieties for the preparation of food flavour [58], which specifies that compounds with aromatic properties or natural fragrance complexes prepared from food by physical, enzymatic, or microbiological methods can be used for food flavours. China’s national standards regulate food additives, food safety, and food labelling, but do not cover specific dosage requirements for particular flavouring ingredients. The experimental results proved that the encapsulation effect was good and effectively protected the presence of multiple volatile components of the same species in coffee. And the use of β-CD and the dosage of compounds are in accordance with GB2760-2014 (China) requirements, which ensures the safety of CD powder.

**Figure 4:**
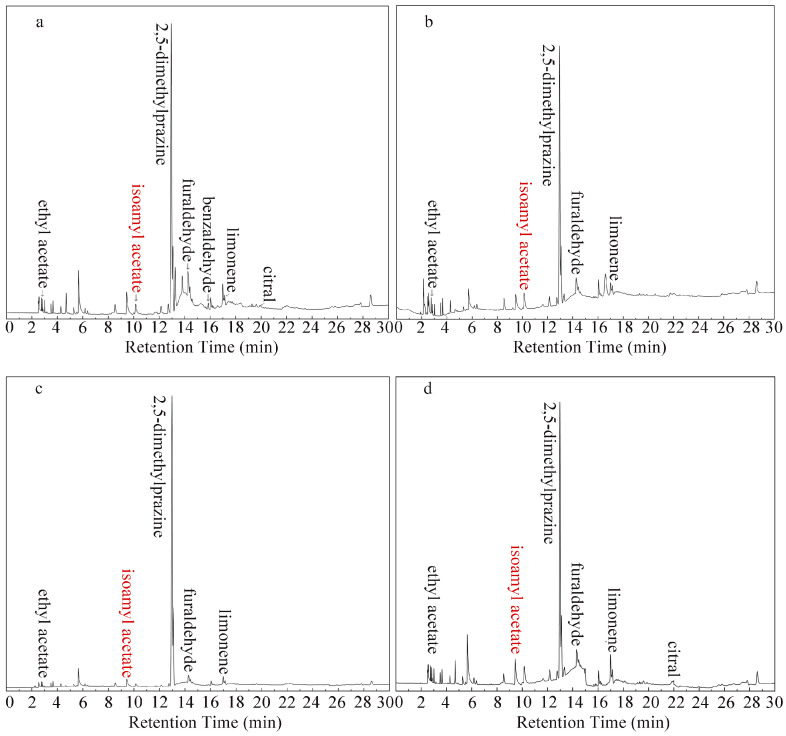
SDE-GC-FID chromatograms for Coffee #1, Coffee #2, Coffee #3, and Coffee #4. (**a**) sinloy yixia (**b**) sinloy landong (**c**) sinloy yishijiaotang (**d**) sinloy mandheling.

SDE-GC-FID was used to detect volatiles in four types of coffee beans. The gas chromatography spectra are shown in Figure 4. According to the results of the analysis, the four coffees do not have the same content and type of volatile compounds. Some of the compounds in the CD powder were clearly detectable in the coffee, such as ethyl acetate, 2,5-dimethylpyrazine, furfural, and benzaldehyde.

The eight volatile compounds and their aroma characteristics in our experiment are shown in Table 3. Linalool and furaldehyde [59,60,61] were detected in odorant compounds of filtered coffee beverages by HS-SPME-GC-MS. Combining with the GC-MS spectral library and NIST (National Institute of Standards and Technology) spectral library, it was found that a high concentration of ethyl acetate, limonene, and benzaldehyde [12,59,60] also existed in the aroma of green coffee beans. Lopes et al. used HS-SPME-GC-MS to analyse the roasted coffee beans and identified phenylethyl acetate in coffee wastewater and coffee pulp [60]. The eight volatile compounds and their aroma characteristics in our experiment are shown in Table 3.

According to the study, aroma is an extremely vital element that defines the quality as well as the level of consumer recognition for coffee products [62,63]. Ethyl acetate can be used as a top note for fresh fruit flavours and is widely used in the formulation of fruit flavours in wine [64]. 2,5-dimethylpyrazine has a roasted peanut aroma and a chocolate, creamy smell. Furfural is mainly used in the formulation of various hot-processed flavours, such as bread, butterscotch, coffee, etc. It is also an important flavouring component in Maotai-flavoured liquor. Limonene is a natural component found in a variety of fruits, vegetables, and spices and is found in high levels in the essential oils of citrus fruits (especially their peels), spices, and herbs [65]. Linalool is widely found in the flowers, fruits, stems, leaves, and roots of plants. It is extremely versatile and can be used not only in all floral flavours, jasmine, lilac, etc., but also in fruity flavours. Citral is found naturally in lemongrass oil (70~80%), sorrel oil (about 70%), citrus leaf oil, etc. [66], and it is widely to enhance the lemon aroma of a variety of foods. Phenylethyl acetate is found naturally in fruits, tea, and rose. It is mainly used as a flavouring component and is indispensable in rose-type flavourings [65]. Cyclodextrins have a wide range of applications, notably in extending shelf life, acting as flavour carriers to minimize flavour loss, and enhancing food flavours as seasoning agents. According to our research, when a compound forms an inclusion complex with cyclodextrin, the resulting complex indeed contributes to a longer product shelf life and can be used as a seasoning agent [67]. 

### 3.3. Enhancing Effect of Coffee and Flavour by β-CD/Flavour CD Powder

**Figure 5:**
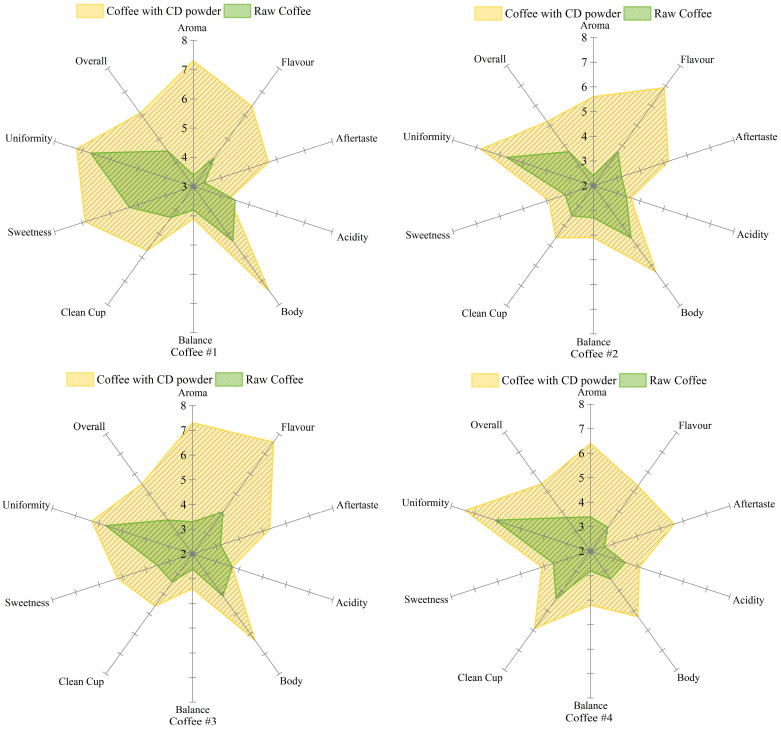
Radar chart comparing before and after scores of CD powders added to coffee samples #1, #2, #3, and #4.

For beverages like coffee, cupping is formally identified as the ultimate test for evaluating its sensory quality [3], even if sensory analysis does have its own shortcomings. In this study, three SCA-certified intermediate baristas and another 50 well-trained coffee tasters were employed to participate in the sensory quality evaluation, and the scores are shown in Figure 5.

Generally, the 53 cupping panellists did not report statistically significant differences between each other. It was found that there were obvious improvements in sensory quality for all four kinds of regular coffee after using CD powder. The aroma and flavour of the coffee was elevated the most. The fruit and flower flavours were clearly perceived by the participants. It is worth noting that there were scarcely any participants who could perceive this kind of aroma and flavour in raw coffee. Similar results could be found for other food usages of β-CD complex [64,65,68,69]. 

In addition, improvements in the aftertaste of coffee were noticed after CD powder was added. This is likely because of the following reasons. On the one hand, studies have found that β-CD can effectively shield compounds from the impact of environmental conditions [70,71], slowly release substances from its cavity [72,73], and improve storage stability [74]. On the other hands, 2,5-dimethylpyrazine has been proven to exist in roasted coffee and is an important component of typical coffee flavour [40]. The cupping test showed that β-CD containing 2,5-dimethylpyrazine could effectively enhance the aftertaste of coffee. Furthermore, nutty, fruity, and floral aromas were also likely to be perceived with the help of β-CD.

**Figure 6:**
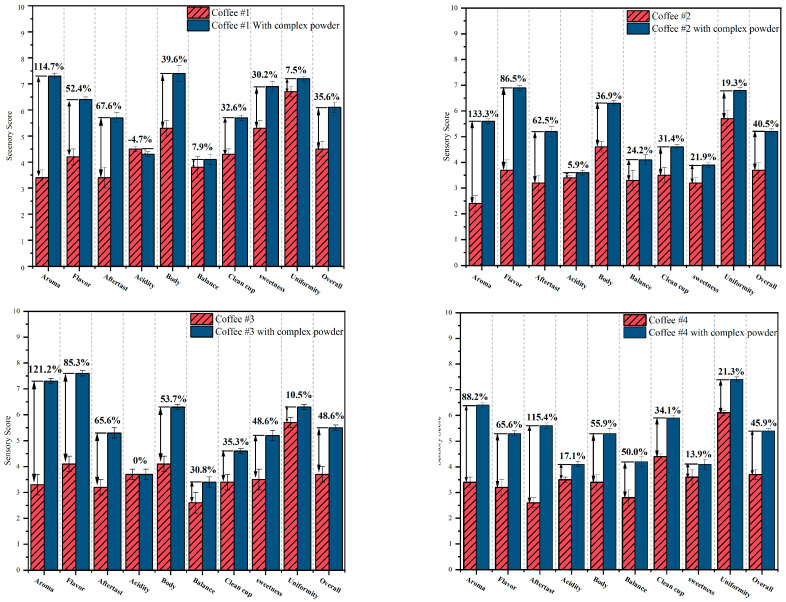
Comparison charts before and after addition of CD powders (Coffee #1, Coffee #2, Coffee #3, and Coffee #4).

Figure 6 shows the comparison charts before and after addition of CD powders to four different original coffees. Compared to the original coffees, the results showed that the sweetness of coffee was up 0.5 to 1.7 points after adding CD powder. This should be related to the nature of the cyclodextrin itself [75]. In terms of uniformity, CD powder works steadily in each cup of coffee and improves the quality of the coffee.

Over the course of a huge number of cupping tests for those four kinds of coffee, CD powder basically has the same effect on each cup of coffee. And the results of sensory evaluation were similar for 53 parallel additions in the same kind of coffee. At the same time, there was no change in uniformity when compared with original coffee. Furthermore, the CD powder had no influence on the aspects of acidity, balance, and clean cup. It is worth noting that the coffee tasted significantly lighter after the inclusion compound was added. 

According to the results of comprehensive analysis and subjective evaluation of flavour, addition of the CD powder to coffee can improve the flavour quality of the coffee to a certain extent. The double-blind experiment further verified the effectiveness of CD powder. All participants in the sensory experiment gave higher scores to coffee with CD powder added without knowing the type of coffee. According to the data counted, it is clear that the standard deviation of sensory evaluation of the original coffee in the double-blind experiment is large, even greater than 1. This indicates that the quality of the original coffee is somewhat controversial and cannot be suitable for most tastes. After the addition of the CD powder, the coffee sensory scores in the double-blind trial were significantly higher, and the standard deviation was significantly smaller. This indicates that the sensory scores obtained with the addition of the CD powder are less discrete. This illustrates the stability, reproducibility, and functionality of CD powder.

**Figure 7:**
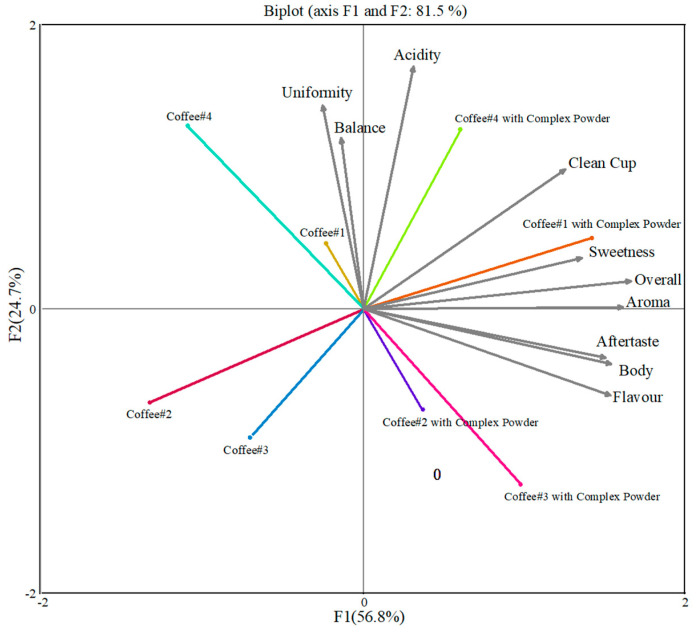
PCA loading plots of the sensory evaluation of coffee samples before and after addition of CD powder.

PCA (principal component analysis) was performed on the sensory data obtained from the coffee cup test using Origin (2021) software, and the results are shown in Figure 7. From the experimental analysis, two principal components were obtained; the first one contributed 56.8%, and the second one contributed 24.7%. The cumulative contribution of the two was 81.5%, which indicates that these indicators of coffee are basically representative of the measured compositional indicators and indicates that these indicators have an important contribution to the sensory flavour of coffee. Figure 7 depicts PCA loading plots of the sensory evaluation of coffee samples before and after addition of CD powder.

The PC2 of the four groups of coffees showed very little or no change after the addition of CD powder, and the PC2 indicated that the consistency, acidity, and balance of the coffees were not affected after the addition of CD powder. The PC1 of the coffee changed very significantly after the addition of the CD powder and was significantly improved. 

The addition of β-CD CD powder significantly increased the PC1 of Coffee #1. The variables ‘Sweetness’, ‘Overall’, and ‘Aroma’ were notably enhanced. Aroma was significantly improved, as β-CD protected and released more aroma compounds. Sweetness was also improved, likely due to β-CD masking bitterness and accentuating sweetness. The overall quality improved, indicating significant enhancement in overall organoleptic attributes. Coffee #2 showed a significant increase in PC1 with the addition of the CD powder, particularly in the ‘Flavour’, ‘Body’, and ‘Aftertaste’ variables. The flavour was significantly improved due to the slow release of flavour compounds from β-CD, enhancing flavour levels. The body became fuller and richer, improving the overall texture of the coffee. The aftertaste was longer and richer, with the slow-release properties of β-CD extending flavour persistence. Coffee #3 also showed a significant increase in PC1 with the addition of the CD powder, especially in the ‘Flavour’, ‘Body’, and ‘Aftertaste’ variables. Flavour was significantly enhanced, with β-CD effectively retaining and releasing flavour compounds. The body was fuller and richer in texture, thanks to the CD powder. The aftertaste was long and rich, with the slow-release properties of β-CD extending the flavour aftertaste.

Coffee #4 shows a significant increase in PC1 with the addition of the complex. Near the “Clean Cup”, “Sweetness”, and “Overall” variables, clean cup was improved, and the CD powder may have enhanced the freshness of the coffee. The coffee has a refreshing flavour. Sweetness increased significantly, with β-CD masking the bitterness. It is the dissolution of the CD powder, the slow release of the flavour compounds, and the effect of cyclodextrin on the taste that led to a greater increase in the first principal component.

### 3.4. Product Stability

When the encapsulation complex is used for the purpose of sustained release, the release behaviour of active substances mainly depends on the affinity between the guest and host molecules over time [76]. Under the specific conditions of this study, the inclusion compounds based on β-CD showed good protective effect on coffee aroma substances and had high stability. In the formation process of CD powder, water must be transferred from the living cavity to be replaced by other guest molecules. Once the inclusion compound is formed, adding water will lead to system failure [77]. So, lower humidity is needed for the store of the CD powder.

**Figure 8:**
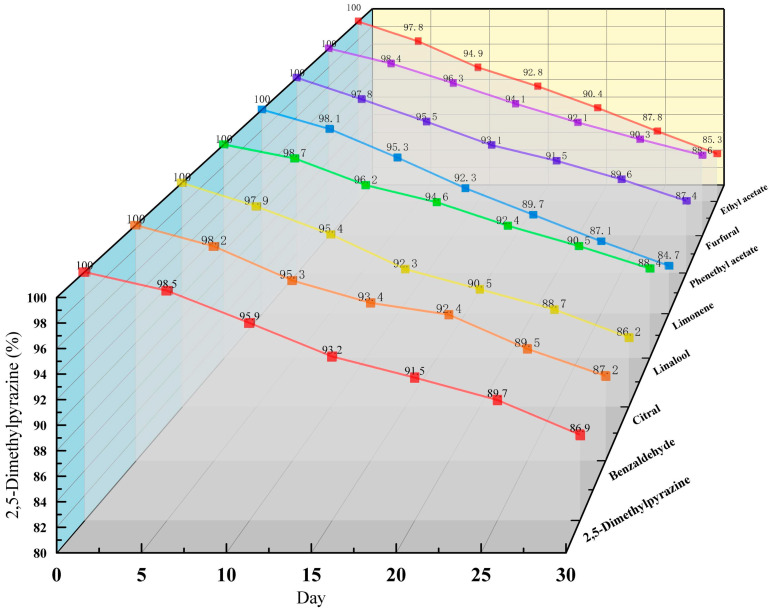
The release curves of different flavour substances.

Figure 8 shows the results of retention rates of inclusion compounds changing with time under the sealed environment of 25 °C. The data showed that the loss rate of the volatile substances in the inclusion complex was less than 20% in one month. It also proved that the inclusion complex has a good embedding effect, strong stability, and good sustained release performance.

### 3.5. Accelerated Destruction Test and Shelf-Life Prediction of β-CD/Flavour CD Powder

In order to assess the shelf life of β-CD/flavour CD powders, the sensory grade of the coffee at different storage durations was selected as an indicator to evaluate taste enhancement and quality improvement.

**Figure 9:**
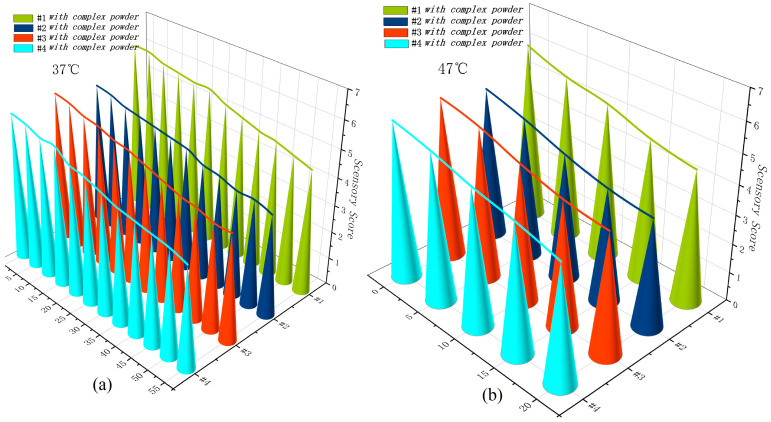
Changes in coffee sensory scores after addition of CD powder stored at 37 °C (**a**) and 47 °C (**b**).

To ascertain the enhancement in coffee quality, it is crucial to observe significant changes in the sensory evaluation of the coffee after the addition of the CD powder. The sensory score of the coffee should not decline below the score of the original coffee after the incorporation of the CD powder, ensuring that the quality of the coffee remains upheld [78]. Figure 9 shows the records of coffee sensory scores after addition of CD powder. It can be seen from Figure 9 that at 37 °C, the shelf life of the product is 55 days, and at 47 °C, the shelf life of the product is 20 days, which can be obtained by using the formula 55/20. The CD powder can be stored for approximately 181 days at 25 °C.

## 4. Conclusions 

Firstly, the β-CD/flavour CD powder was prepared by utilizing saturated aqueous solution and freeze-drying techniques. The formation of the CD powder was further confirmed using FT-IR, XRD, and SDE-GC-FID techniques, which established the presence of eight compounds within it. These compounds included two esters, three aldehydes, one alcohol, one alkene, and one pyrazine. 

Subsequently, sensory evaluations demonstrated a significant improvement in the quality of the original coffee with the addition of the β-CD/flavour CD powder. In cup testing experiments, the CD powder exhibited pronounced enhancements in aroma, flavour, aftertaste, cup cleanliness, sweetness, and overall rating. Notably, aroma scores experienced an increase of 3.0–4.0 points, while flavour scores improved by 2.1–3.5 points. Importantly, the CD powder did not affect the acidity, balance, and uniformity of the coffee. Finally, the β-CD/flavour CD powder exhibited exceptional stability and storage performance in its powdered state. Through accelerated degradation testing, it was determined to have a shelf life of 181 days at 25 °C, making it convenient for transportation, storage, and usage. Therefore, compared to traditional coffee bean processing methods, the CD powder offers numerous benefits while ensuring safety. From a supply chain perspective, particularly concerning long-distance transportation and extended storage, the CD powder’s ability to significantly extend the shelf life of coffee products will have positive implications for supply chain management, such as reducing inventory losses and improving logistics efficiency. For manufacturers, this means that they can leverage this technology to enhance coffee quality in terms of aroma and taste at a relatively low cost, catering to diverse consumer segments across different price points. This broadens the customer base and market coverage, potentially enabling the creation of distinctive coffee product lines for competitive differentiation.

## Figures and Tables

**Table 1:** Column temperature ramp-up progress.

Time (min)	Temperature (°C)	Rate (°C min^−1^)	Action
0.0	30	0.0	Initial temperature
2.0	30	0.0	Maintain for 2 min
8.0	33	0.5	Ramp up and maintain
9.0	33	0.0	Maintain for 1 min
12.5	40	2.0	Ramp up and maintain
13.5	40	0.0	Maintain for 1 min
18.5	90	10.0	Ramp up and maintain
19.0	90	0.0	Maintain for 0.5 min
21.0	100	5.0	Ramp up and maintain
21.5	100	0.0	Maintain for 0.5 min
25.5	180	20.0	Ramp up and maintain
26.0	180	0.0	Maintain for 0.5 min
27.8	230	30.0	Ramp up and maintain
29.8	230	0.0	Maintain for 2 min

**Table 2:** A list of volatile compounds detected in CD powder and their respective concentrations.

No.	Retention Time/min	Name	Proportion/%
1	2.9	Ethyl acetate	0.98%
2	12.9	2,5-dimethylprazine	0.87%
3	14.1	Furaldehyde	1.58%
4	15.8	Benzaldehyde	0.55%
5	17.2	Limonene	0.64%
6	17.6	Linalool	0.62%
7	19.9	Citral	0.71%
8	25.1	Phenylethyl acetate	0.57%
9	———	β-CD	93.48%

**Table 3:** A list of volatile compounds detected in coffee and their aroma characteristics with corresponding China national standards.

No.	Name	Flavour	China National Standards	References
1	Ethyl acetate	Cherry, peach, apricot	GB2760—2014-S0364	[8,49,56,57]
2	2,5-dimethylprazine	Roasted peanut, chocolate, creamy smell	GB2760—2014-S0712	[52]
3	Furaldehyde	Almond oil	GB2760—2014-S0180	[55,56,57]
4	Benzaldehyde	Bitter almonds, cherries, nuts	GB2760—2014-S0165	[8,56,57]
5	Limonene	Citrus fruits	GB2760—2014-S0654	[8,51,56,57]
6	Linalool	Bergamot	GB2760—2014-S0029	[55,56,57]
7	Citral	Lemon	GB2760—2014-S0174	[48]
8	Phenylethyl acetate	Apples, grapes, poplar berries, tea, timbrel, rose	GB2760—2014-S0383	[50,57]

## Data Availability

The original contributions presented in the study are included in the article, further inquiries can be directed to the corresponding author.
